# Regulation of P-Glycoprotein during Oxidative Stress

**DOI:** 10.3390/antiox13020215

**Published:** 2024-02-08

**Authors:** Aleksey V. Shchulkin, Yulia V. Abalenikhina, Olga V. Kosmachevskaya, Alexey F. Topunov, Elena N. Yakusheva

**Affiliations:** 1 Pharmacology Department, Ryazan State Medical University, 390026 Ryazan, Russia; abalenihina88@mail.ru (Y.V.A.); e.yakusheva@rzgmu.ru (E.N.Y.); 2 Bach Institute of Biochemistry, Research Center of Biotechnology, Russian Academy of Sciences, 119071 Moscow, Russia; rizobium@yandex.ru (O.V.K.); aftopunov@yandex.ru (A.F.T.)

**Keywords:** P-glycoprotein, oxidative stress, reactive oxygen species, Nrf2, Nf-kB

## Abstract

P-glycoprotein (Pgp, ABCB1, *MDR1*) is an efflux transporter protein that removes molecules from the cells (outflow) into the extracellular space. Pgp plays an important role in pharmacokinetics, ensuring the absorption, distribution, and excretion of drugs and its substrates, as well as in the transport of endogenous molecules (steroid and thyroid hormones). It also contributes to tumor cell resistance to chemotherapy. In this review, we summarize the mechanisms of Pgp regulation during oxidative stress. The currently available data suggest that Pgp has a complex variety of regulatory mechanisms under oxidative stress, involving many transcription factors, the main ones being Nrf2 and Nf-kB. These factors often overlap, and some can be activated under certain conditions, such as the deposition of oxidation products, depending on the severity of oxidative stress. In most cases, the expression of Pgp increases due to increased transcription and translation, but under severe oxidative stress, it can also decrease due to the oxidation of amino acids in its molecule. At the same time, Pgp acts as a protector against oxidative stress, eliminating the causative factors and removing its by-products, as well as participating in signaling pathways.

## 1. Introduction

P-glycoprotein (Pgp, ABCB1 protein, *MDR1*) is a transporter protein, the most studied representative of the ABC transporter superfamily [1]. Pgp is an efflux protein: it ensures the outflow of molecules from cells into the extracellular space. The main function of Pgp is excreting xeno- and endobiotics into the extracellular space, biological fluids (blood, bile and urine) and intestinal lumen. Pgp plays an important role in pharmacokinetics—absorption, distribution, and excretion of drugs, which are its substrates; is involved in the transport of endogenous molecules (steroid and thyroid hormones); and contributes to the resistance of tumor cells to chemotherapy [2,3,4,5]. However, the hypothesis that the sole function of Pgp is to remove xenobiotics from the cell does not explain the high level of the transporter protein in the adrenal glands [6] or its apical localization in the epithelial cells of the vascular plexus [7]. Pgp is also able to block the development of apoptosis in tumor cells by modulating the activity of key enzymes for their programmed death [8,9]. Thus, Pgp plays a key role not only in the pharmacokinetics of drugs, being its substrates, but also in physiological and pathological processes.

The activity of Pgp can vary significantly under the influence of the external and internal environmental factors, such as genetic characteristics of the body, oxygen concentration in blood, acid–base balance, the use of a number of drugs, etc. [10]. According to modern concepts, Pgp activity can change as a result of the following main processes: modulation of the expression of the multidrug resistance gene (*MDR1*, multidrug resistance gene) and the activity of the synthesized protein [11,12]. At the same time, the activity of Pgp can both decrease (inhibition) and increase (induction) [13,14]. That is why the regulation mechanisms of the transporter protein are currently being actively studied.

Studies from the early 1960s to the 1980s showed that the increased production of reactive oxygen species (ROS) plays an important role in the pathogenesis of the most common human diseases (pathology of the cardiovascular, respiratory, endocrine systems and cancers) [15,16,17]. Mainly lipid and protein molecules are subject to free radical oxidation in cells [18,19,20,21]. The free radical oxidation of biomolecules leads to oxidative stress (OS), characterized by highly toxic oxidation products accumulated in blood and tissues [20]. Oxidative modification of both lipids and proteins changes the viscosity, elasticity and fluidity of membranes, which significantly affects the cell–cell interaction, mitosis and endocytosis [22]. Therefore, a number of researchers consider the cell membrane as a biosensor of the OS [23].

Pgp is localized not only in plasma membranes but also in the membranes of intracellular compartments (endoplasmic reticulum, Golgi apparatus, lysosomes and mitochondria), and the role of the transporter in these compartments continues to be studied [24,25]. Free radical oxidation of membrane lipids in different compartments may disrupt the activity of Pgp. That is why the etiology of OS development (exogenous or endogenous formation of ROS) is important in studying the regulation of Pgp activity and expression. In addition, the peroxidation products, formed as a result of OS, may directly or indirectly affect the functioning of Pgp or its substrates. The functioning of the transporter protein is of great practical importance due to the role of Pgp in the pharmacokinetics of a wide range of drugs, as well as its contribution to the development of pharmacotherapy-resistant diseases (epilepsy and tumors). In this regard, studying OS’s effect on the activity of Pgp will significantly improve the effectiveness and safety of the ongoing therapy by predicting pharmacokinetics and adverse drug reactions.

In contrast to similar reviews [26], the main focus in this work is on the mechanisms of Pgp regulation under OS, the possible reasons for the inconsistency of results obtained in different studies, and on the role of Pgp in cell protection during OS rather than in the pathogenesis and treatment of various diseases.

## 2. Pgp: Localization, Structure, Inhibitors and Substrates and Mechanisms of Regulation

### 2.1. Brief History and Overview of Pgp

Pgp was first isolated in 1976 by Juliano and Ling from the plasma membrane of Chinese hamster ovarian cells, selected for colchicine resistance and demonstrating cross-resistance to a wide range of amphiphilic substances [27]. The protein was named P-glycoprotein (P—permeability) because it was believed to be involved in the development of drug resistance via reducing the permeability of the cell membrane [28]. Ten years later, the genes responsible for multidrug resistance (*MDR* genes) were cloned in humans, mice and hamsters [29,30,31], and Pgp was shown to be the protein product of the *MDR1* gene [32]. Subsequently, Fojo et al. (1987) demonstrated that the gene encoding Pgp—*MDR1* in humans—is highly expressed in the adrenal glands and kidneys, at an average level in the lungs, liver, jejunum, colon and rectum (samples were obtained by paracentesis) [33].

Thiebaut et al. (1987) immunohistochemically studied Pgp localization in human tissues obtained during autopsy or surgical operations [34]. They showed that Pgp is localized in the liver, mainly on the biliary surface of hepatocytes and on the apical surface of epithelial cells in small biliary ducts. In the pancreas, Pgp was found on the apical surface of epithelial cells of small ducts, but not of large ones. In kidneys, it was found on the apical surface of the epithelial cells of the proximal renal tubules. Colon and jejunum showed high levels of Pgp on the apical surface of epithelial cells. The adrenal glands were characterized by high levels of Pgp on the cell surface in the medulla and cortex [34]. In 1994, Schinkel et al. revealed the expression of *mdr1a* in the endothelial cells of the capillaries in the blood–brain barrier [35]. Its expression was also found in cells on the luminal surface of the endometrium of the pregnant uterus, and also in placental trophoblasts [36].

Initially, Pgp was supposed to protect tumor cells from the effect of cytostatics by removing them from the cells [37]. However, it was later shown that Pgp performs an important function of transporting endogenous substances and also protects cells from the effects of xenobiotics, providing their efflux into the extracellular space and biological fluids (blood, bile and urine) [38,39]. In the blood–brain barrier, Pgp protects the brain from exposure to toxic substances. In mice with Pgp gene knockout, the concentration of xenobiotic in the brain increased 20–80 times, while the levels of xenobiotics in other organs, such as liver, kidneys and intestines, increased only 2–4 times [35]. Compared to the wild-type mice, mice with Pgp gene knockout require a lower dose of the drug pilocarpine to induce seizures [40]. In mammalian kidneys, Pgp ensures the excretion of drugs, their conjugates and metabolites into the lumen of the proximal renal tubules [41]. For example, in the canine kidney, tubule cells of Madin Darby (MDCK) transfected with the *ABCB1* gene encoding Pgp, the basolateral to apical outflow of the oncological drug gefitinib, was significantly increased compared to the corresponding control cells. In the presence of Pgp inhibitor LY335979, the outflow of gefitinib in cells transfected with the *MDR1* gene decreased to the same level as in the control cells [42]. 

In hepatocytes, Pgp is expressed on the apical (biliary) surface, ensuring the excretion of xenobiotics into bile. Furthermore, Pgp inhibition can also affect bile excretion [43,44,45,46]. In enterocytes, Pgp is expressed on the apical surface and prevents the uptake of substrates into the systemic blood flow [46,47,48]. 

**Figure 1:**
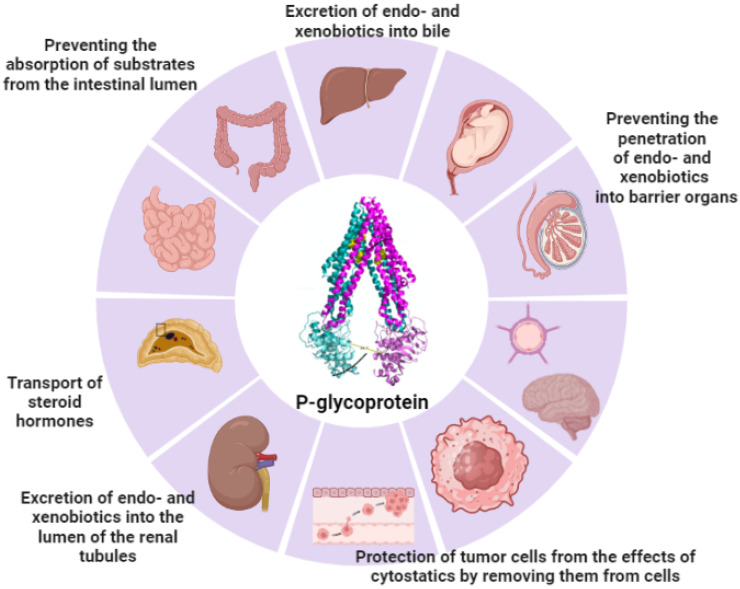
Localization and functions of Pgp (the figure was made using app.biorender.com, accessed on 2 October 2023).

Pgp transports a wide range of structurally and functionally different cytotoxic compounds out of the cell. Overexpression of Pgp in cancer cells, compared to normal cells, is the main cause of multidrug resistance (MDR). Therefore, the inclusion of Pgp inhibitors in drugs can reduce Pgp activity in cancer cells [49] and thereby increase therapeutic efficacy [50]. Pgp is an essential component in the drug distribution process, contributing to drug–drug interactions [51] (Figure 1).

### 2.2. Structure of Pgp and Its Substrates and Inhibitors

Pgp is an N-glycosylated transmembrane glycoprotein that weighs 170 kDa. It consists of 1280 amino acid residues in humans and of 1276 amino acid residues in mice (bears 87% sequence identity to human Pgp) grouped into two homologous halves, which are connected to each other by a mobile linker polypeptide (Supplementary Materials) [52,53].

**Figure 2:**
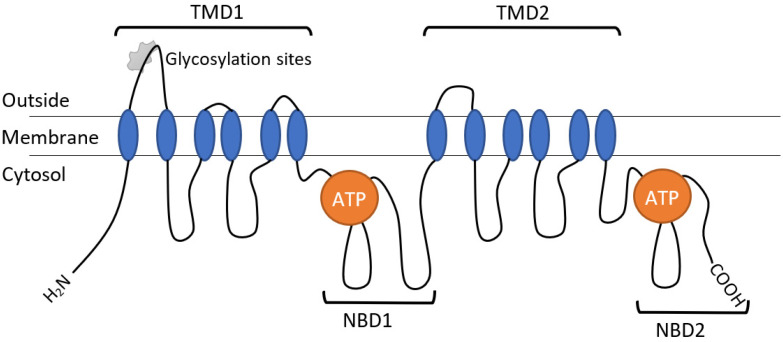
Schematic representation of the structure of the human Pgp transporter protein.

Each part consists of an NH2-terminal transmembrane domain (TMD) containing six transmembrane (TM) segments (the so-called hydrophobic segments or α-helices) interconnected by several intracellular and extracellular loops (hydrophilic loops), as well as a hydrophilic (intracellular) domain containing ATP-binding sites (NBDs), which are placed inside the plasma membrane. Analyzing the secondary structure of Pgp showed that the transporter protein consists of 32–43% α-helices, 16–26% β-folded layers (β-structures, β-sheets), 15–29% β-bends and 13–26% irregular secondary structures (Figure 2) [54,55,56].

ATP energy is necessary for the Pgp operation and for the stabilization of its external conformation [57,58].

Currently, the tertiary structure of a multidrug-resistant protein remains unclear, mainly due to the difficulties associated with protein crystallization. The tertiary structure of Pgp is known to be highly flexible, providing for three-dimensional reorientation. This characteristic of Pgp further facilitates its interaction with a wide range of substrates [59,60].

To enter the hydrophobic binding pocket of the Pgp and pass through the membrane, the molecule must be lipophilic. Free energy calculating showed that hydrophobic fragments mainly contribute to ligand binding. Many studies suggest that the hydrophobicity of the molecule is crucial to the development of transporter inhibitors. On the contrary, the hydrophilicity of the molecule may interfere with Pgp efflux [61].

Nonpolar, linear, hydrophobic and aromatic compounds with different molecular weights, ranging from 250 to 4000 Da, were identified as Pgp substrates [62,63]. The Pgp substrates are sphingolipids; platelet activation factor; short-chain phospholipids; cytokines; and lipophilic drugs—antitumor drugs, immunosuppressants, steroid and thyroid hormones, antibiotics, HIV proteinase inhibitors, cardiac glycosides, anticoagulants, etc. [10,51]. 

Using computational methods and programming, combined with structural design [64], the necessary characteristics of inhibitors that are potentially suitable for clinical use were determined:

(a) A high log *p*-value (determined by the lipid and water distribution coefficient for the drug). This parameter for the compound must be at least 2.92 or higher, which is necessary for the formation of a hydrophobic/van der Waals interaction with the Pgp binding site;
(b) Significant molecular weight—A molecule must have 18 or more atoms to cover more than one Pgp binding site;
(c) The energy of the highest occupied molecular orbital (highest occupied molecular orbital, HOMO) should have a higher value to ensure the nucleophilic interaction of the molecule with Pgp;
(d) At least one tertiary nitrogen atom since the tertiary amine forms a cation at physiological pH and guarantees binding by ionic interaction.

Many studies report on the aromatic rings; molecular weight; cationic charge, such as protonated amine; and H-bond donor/acceptor factors. In general, inhibitors have a higher log *p*-value than substrates. These inhibitors act primarily as H-bond donors rather than as H-bond acceptors. Functional groups, including arene, alkyl, carbonyl, ether and nitrogen ones, are necessary for strong interactions between the protein and the inhibitor, thereby affecting the effectiveness and pharmacodynamic aspect of the interactions. Studies on the dependence of structure and activity have shown that lipophilicity and the value of log ligands (substrate and inhibitor) are important parameters affecting pharmacokinetic aspects [65,66]. 

Pgp inhibition can also occur as a result of changes in its structure. For example, flavonoids [67], xanthones [68] and synthetic taxoids (derivatives of the antitumor agent taxol) cause conformational changes in Pgp, and such changes, in turn, disrupt the outflow function due to the formation of H-bonds and ATP hydrolysis [69].

### 2.3. Mechanisms of Regulation of Pgp

The following mechanisms of Pgp regulation are distinguished.

(1) Mechanisms of the *MDR1* gene expression regulation.

Pgp in humans is encoded by the *MDR1* gene, while in mice and rats, it is encoded by the *mdr1a* (also called *mdr3*) and *mdr1b* (also called *mdr1*) genes, and the homologous halves of Pgp are the product of duplication of the *MDR1* gene [35,70,71]. It was previously assumed that, in humans, Pgp can also be encoded by the *MDR3* gene (sometimes referred to as *MDR2*) and the *mdr2* gene in mice. However, these genes were later shown not to be involved in multidrug resistance and to encode phospholipid flippase expressed in the biliary membranes of hepatocytes. They were revealed to play an important role in the secretion of phosphatidylcholine into bile. According to the international classification, this gene is designated as *ABCB4* [70]. An increase in the expression of the *MDR1* gene increases Pgp activity, and a decrease in its expression leads to its decrease. The *MDR1* gene can be transcribed from two promoters—distal and proximal [71,72]. Proximal promoter regulates the expression of *MDR1* in normal tissues, including the liver, kidneys and adrenal glands, while the distal promoter triggers the expression of *MDR1* in colchicine-selected (selected) cells [33,73], mononuclear cells of patients with acute lymphoblastic leukemia who overexpress *MDR1* [74], and cells of the primary breast tumors [75]. The effect of the nucleotide sequence of the *MDR1* gene promoter on its transcription and expression was studied using luciferase as a reporter of this gene. The expression of the *MDR1* gene can be influenced by both environmental factors and chemicals [76].

(2) Polymorphism of the *MDR1* gene.

Screening of the *MDR1* gene revealed about 50 substitutions of one nucleotide for another, the so–called polymorphisms of one nucleotide—SNP (single-nucleotide polymorphisms) [77,78]. Most polymorphisms of the *MDR1* gene do not significantly affect the expression and functional activity of Pgp [79,80]. Recently, analyzing whole haplotypes rather than the isolated polymorphisms of a single nucleotide was proposed [81].

(3) Increasing the dose of the gene—amplification of a section of the genome containing the *MDR1* gene.

Kitada et al. revealed an increase in Pgp activity due to an increase in the dose (amplification) of the *MDR1* gene. When treating the PTX250 lung cancer cell line with paclitaxel, the number of *MDR1* gene copies was shown to increase 11 times, while the amplicon size was 2.7 megabytes [82]. It was also found that lung cancer cell sublines No. 15-80-1 and No. 15-80-6, which are obtained when treating the NCI-H460 cell line with increasing doses of the inducer Pgp paclitaxel (from 50 to 800 nM), are characterized by amplification of the *MDR1* gene with a different number of copies. However, a common pattern of amplification is accompanied by an increase in Pgp activity [82].

(4) Stabilization of the mRNA of the *MDR1* gene.

The Pgp activity may increase in the background of the stabilization of the mRNA of the *MDR1* gene. For example, in myelogenous leukemic K562 cells, the level of *MDR1* gene mRNA was found to be independent of the transcriptional activity, but it was regulated at posttranscriptional stages: mRNA stability and translational regulation. In particular, the short mRNA life of the *MDR1* gene of native cells (1 h) increased to 12–16 h after short-term exposure to Pgp inducers—colchicine and doxorubicin [83]. In another study, it was shown that, during the treatment of T-cell leukemia cell culture with cytostatic cytarabine for 36 h, the half-life of the mRNA of the *MDR1* gene increased from 30 min to 6 h [83]. At the same time, an increase in the stability of Pgp mRNA was accompanied by an increase in the amount of Pgp protein only with the long-term selection of cytostatic-resistant cells, and the short-term stabilization of mRNA did not lead to an increase in Pgp synthesis and activity. These results indicate that cell resistance to cytostatics is associated with two processes: stabilizing the mRNA of the *MDR1* gene; and the overcoming of translational block, which is necessary to start Pgp synthesis [84].

(5) The effect of microRNAs on Pgp expression.

Several microRNAs were described as direct regulators of Pgp expression [85]. For example, microRNA-451 was shown to regulate Pgp expression through direct interaction with the 3′-untranslated region (3′-UTR) of *MDR1* mRNA. An increase in the content of microRNA-451 in the doxorubicin-resistant breast cancer cell line (MCF-7-DOX) led to a decrease in Pgp expression and, more importantly, to an increase in cellular sensitivity to doxorubicin [86]. An inverse correlation between the levels of Pgp and microRNA-331-5p was found. It was additionally shown that overexpression of microRNA-331-5p increases the sensitivity of K562-DOX cells to doxorubicin by inhibiting Pgp expression [87]. Impaired microRNA-298 formation (due to low expression of the Dicer enzyme) was associated with modulating Pgp expression. MicroRNA-298 was shown to bind directly to the 3′-untranslated region (3′-UTR) of the mRNA of the *MDR1* gene. The decreased level of this microRNA was accompanied by an increase in Pgp expression and the development of doxorubicin resistance in breast cancer cells (MDA-MB-231) [88]. In another study on a human colon carcinoma cell line (Caco-2), a decrease in the content of microRNA-145 increased the expression and activity of Pgp, but not the amount of mRNA of the *MDR1* gene, indicating that, in this cell line, microRNA-145 regulates Pgp through translational mRNA repression [89]. Several microRNAs were described as “indirect regulators” of Pgp. These microRNAs interacting with mRNAs encoding intermediate proteins or transcription factors indirectly participate in activating the *MDR1* gene [85]. For example, microRNA-let-7 was shown to regulate *MDR1* in ovarian cancer cells by suppressing IMP-1, an RNA-binding protein, which destabilizes *MDR1* mRNA and, as a consequence, decreases Pgp expression [90]. This indicates the possibility of indirect influence of these microRNAs on the protein transporter [91].

(6) Pgp transmission between cells.

All cells, prokaryotes and eukaryotes, release extracellular vesicles as part of their normal physiology and during acquired abnormalities. Depending on the cell of origin, extracellular vesicles can contain many constituents of a cell, including DNA, RNA, lipids, metabolites, and cytosolic and cell-surface proteins [92]. The ability of the Adriamycin-resistant culture of bladder cells (BIU87) to transmit Pgp to sensitive cells by using microparticles of the plasma membrane (microparticles) was revealed. At the same time, different cell cultures were incubated for 48 h in media, separated by a membrane that prevented their direct contact. The presence of Pgp on the recipient cell membranes was determined via the Western blot method, and its functioning was confirmed by the active efflux of rhodamine-123, a substrate of the transporter protein [93]. In vitro and in vivo studies showed that the transfer of protein transporter can occur between cells of various origins: tumor and unchanged; and human and mouse. It was revealed that Pgp is transported via binding to microparticles of plasma membranes larger than 0.8 microns in size, while there is no cell fusion or formation of gap contacts between them [94]. Pgp transmission to sensitive tumor cells inhibits their growth and forms an unstable resistant state. It allows them to survive in an environment containing high doses of chemotherapeutic agents for a time, sufficient to acquire their own resistance due to the independent expression of Pgp [94].

(7) Changes in the activity of the synthesized transporter protein.

The activity of Pgp can change as a result of the direct interaction of the transporter protein molecule with molecules of endogenous and exogenous substances. Meanwhile, Pgp activity can both decrease (inhibition) and increase (induction) [95,96]. To date, three ways to inhibit the transporter protein activity have been described: competitive, noncompetitive and allosteric [97]. In competitive and noncompetitive inhibition, the inhibitor interacts with binding centers of substrates, and in the allosteric one, with the allosteric center. Data on these centers were summarized in papers by Martin et al. (2000) [98], Ferreira et al. (2013) [99] and Bocci et al. (2018) [100]. The authors identified three binding sites of substrates (transported substances) and one regulatory (allosteric, M-site) site that changes the activity of transporter protein. The three sites of substrate binding were named the D, R and H sites, according to the names of bound substrates—digoxin, rhodamine-123 and hext-33342, respectively. The competitive mechanism of Pgp activity inhibition assumes that the inhibitor binds to one, two or three sites and inactivates them. At the same time, if there are free binding sites, they can transport their substrates. For example, terfenadine is capable of inhibiting the transfer of Pgp hext-33342 and rhodamine-123, but not that of digoxin. Prazosin blocks the transport of digoxin and hext-33342, but not that of rhodamine-123. On the contrary, some other compounds, such as reserpine and loperamide, interfere with the transport of three Pgp substrates [100,101].

(8) Effect on ATP hydrolysis (ATPase activity).

Pgp is an ATP-dependent transporter protein, implicating ATP energy at the cornerstone of its functioning. In a study on cells overexpressing the protein, it was found that sodium azide—an inhibitor of metabolic processes—reduces its activity [102]. Some flavonoids (e.g., quercetin) also reduce Pgp activity by inhibiting ATPase activity of the protein transporter. Their advantage is that they do not compete with substrates for binding centers and inhibit the transporter protein at lower concentrations [97].

(9) Changes in plasma membrane characteristics.

Many surfactants, such as sodium dodecyl sulfate, Tween-20 and Span-80, change the characteristics of membrane lipids and, as a consequence, the fluidity (viscosity) of membranes, which, in turn, can inhibit Pgp activity [103]. A change in the microviscosity of the membrane can contribute to a change in the conformation of most transmembrane proteins. For example, the modification of the secondary and tertiary structure by surfactants was shown to decrease Pgp activity [97]. Recently, other substances inhibiting Pgp by increasing membrane fluidity were described, for example, a new synthetic derivative of rifampicin, 1,8-dibenzoylrifampicin, formed by introducing hydrophobic substituents into a positively charged rifampicin molecule. Although this substance has not yet been tested in vivo, it may be an interesting strategy for increasing the therapeutic activity of Pgp substrates [104]. 

Among all mechanisms of Pgp regulation, changes in the expression of the *MDR1* gene and changes in the activity of the synthesized transporter protein are of paramount importance.

## 3. Contemporary Insights into Oxidative Stress

### 3.1. The Concept of Oxidative Stress

In intact cells, the production of ROS is in a subtle equilibrium with antioxidant protection systems. An imbalance towards prooxidants in the cell leads to the ROS-dependent damage of macromolecules (proteins, lipids, carbohydrates and nucleic acids). Antioxidant protection does not completely eliminate ROS, but it controls their levels, and this may be due to the following reasons [15,105,106]:

(1) To maintain excessive antioxidant protection, additional energy will be required, so it may be energetically “cheaper” to restore or replace damaged biomolecules;
(2) Antioxidants may be unable to intercept some ROS, such as •OH;
(3) ROS are necessary for maintaining vital activity since they contribute to launching the intracellular signaling pathways. Antioxidant protection minimizes the levels of most ROS, at the same time, leaving them in a sufficient quantity to perform signaling functions.

The oxidative stress (OS) concept was formulated in 1985 [16]. OS is understood as “an alteration of the prooxidant-antioxidant balance in favor of oxidants” [107]. At that time, the main focus of scientific research was on oxidative damage to cells and organs and the study of various prooxidants and antioxidants [107]. In the following decades, a large number of fundamental works on redox regulation and signaling were performed. This required updating the OS concept [108,109], thus defining it as “an imbalance between oxidants and antioxidants in favor of the oxidants, leading to a disruption of redox signaling and control and/or molecular damage”. 

It follows from this definition that OS can be classified by intensity; and the intensity scale varies from physiological OS (eustress) to toxic oxidative load, damaging biomolecules (distress) [110,111,112].

Low-intensity exposure is used to transmit redox signals by acting on specific targets, whereas high-intensity exposure disrupts regulatory mechanisms and/or damages cells [110,113].

**Figure 3:**
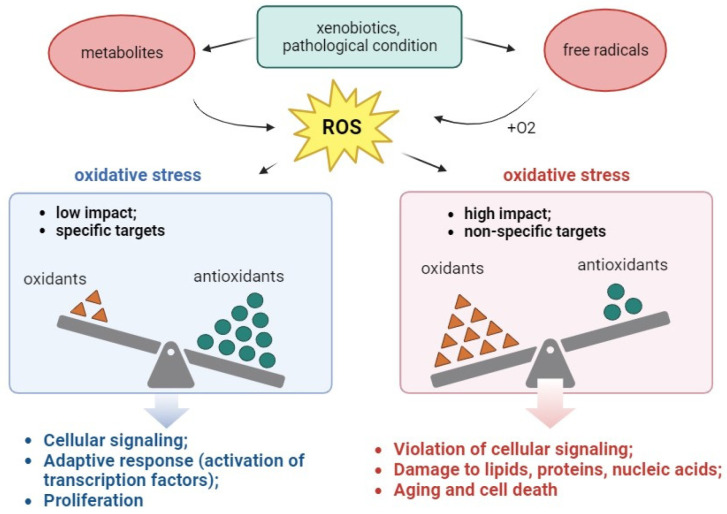
Causes and consequences of OS for a living system.

The consequences of OS may include any of the following phenomena or any combination of them, depending on the type of cell/tissue, as well as the severity and duration of OS [15]:

(1) Increased proliferation—Many cells respond to moderate OS by cell division;
(2) Adaptation of a cell or an organism or the acquisition of resistance to higher levels of OS as a result of activated protective systems;
(3) Cell damage—Individual or all molecular targets are oxidized: proteins, lipids, carbohydrates, nucleic acids. Noteworthy, the damage caused by OS can be primary (direct interaction of ROS with targets) and secondary (interaction of lipid peroxides with amino acid residues of proteins);
(4) Aging—The cell survives, but it can no longer divide;
(5) Cell death—Oxidative damage, especially that of DNA, can cause death as a result of apoptosis or necrosis (Figure 3).

### 3.2. Targets of Oxidative Damage

The main components of biological membranes are lipids and proteins. As a result of OS in most biological membranes, both membrane proteins and lipids are damaged [15,114].

Polyunsaturated fatty acids (PUFAs) are more sensitive to ROS. Of note, saturated and monounsaturated lipids can also be oxidized, but this process is slower and more complicated [18,19,20], and PUFA themselves, as a result of oxidation, can be a source of lipid mediators [115].

In general, lipid peroxidation (LP) reduces the fluidity of the membrane; facilitates the exchange of phospholipids in the bilayer; increases the “permeability” of the membrane for substances that normally do not penetrate the lipid bilayer; damages membrane proteins; and inactivates receptors, enzymes, transport proteins, etc. One of the reasons for these changes is that peroxyl radicals and peroxides are more polar than the inside of normal membranes and therefore change the structure, as they “tend” to be closer to water [116].

Further oxidation of fatty acid chains and their fragmentation with the formation of aldehydes and hydrocarbons disrupts membrane integrity. For example, the rupture of lysosomal membranes releases hydrolytic enzymes and iron ions into the cell. As a result of the peroxidation of erythrocyte membranes, they lose the ability to change their shape and penetrate through small capillaries [117]. Oxidative damage to the endoplasmic reticulum or Golgi apparatus reduces the ability of cells to glycosylate and export proteins [118,119,120].

Together with lipids, protein molecules undergo oxidation. Protein damage during OS can occur as a result of the following:

1. Direct exposure to free radicals (the most reactive •OH);
2. Producers of ROS (e.g., HOCl and ONOOH);
3. “Secondary damage”, including reaction with the final products of LP, such as isoketals, malondialdehyde (MDA) and hydroxynonenal [121,122].

Peroxyl and alkoxyl radicals, aldehydes and some other peroxidation end products can damage receptors and enzymes such as glucose-6-phosphatase, Ca^2+^-ATPase of the endoplasmic reticulum, Na^+^-, K^+^-ATPase and K^+^ channels [123,124,125,126].

Some “damage” to proteins is reversible, for example, the formation of disulfide bonds, sulfenic acid, methionine sulfoxide and S-nitrosylation and the destruction of Fe-S clusters under the action of oxidants [126,127]. Other types of damage, such as the oxidation of the side chains of amino acids or the cleavage of the peptide backbone, can be irreversible, and then the protein undergoes proteolysis [128]. The oxidative damage of DNA (through mutation) and RNA (incorrect transcription) can also lead to the accumulation of defective proteins and be a marker in pathology [129,130].

In addition, OS can interfere with protein synthesis by affecting initiation factors, e.g., initiation factor 2, which is inactivated by phosphorylation caused by OS. Inactivation of factor 2 initiation regulates the reactions of the endoplasmic reticulum to stress. This mechanism helps to reduce the overall protein synthesis under OS conditions, so as not to “flood” the cell with damaged proteins. It possibly gives time for reprogramming the protein synthesis to replenish heat shock proteins, antioxidant enzymes, etc. [131,132].

Oxidative protein modification can result in damage to specific amino acid residues, the formation of new antigens and the fragmentation and loss of enzymatic function. It can also result in conformational changes, leading to the exposure of hydrophobic residues on the surface of the protein and varying degrees of aggregation, which is partly facilitated by the appearance of surface hydrophobic sites. Under OS conditions, the structure and characteristics of the plasma membrane change. Protein and lipid oxidation leads to a change in the viscosity, elasticity and fluidity of the membranes; a lower density of intercellular contacts; and, as a consequence, a decrease in Pgp functioning. A characteristic feature of OS is the launch of the stress response, implemented through signaling mechanisms inducing gene expression of the protective systems activated to restore the redox balance. Under OS conditions, transcription factors such as Nf-kb, Nrf2, HIF and sp1 and 3 can be activated, which theoretically can bind to the promoter of the *MDR1* gene and cause a change in Pgp expression. Thus, under these conditions, membrane and cytoplasmic lipids and proteins are oxidized, which affects Pgp functioning. LP products, as signaling molecules, oxidatively modify amino acids and proteins and act as tools for regulating Pgp functioning. Pgp regulation is a complex multistep process occurring through different mechanisms. Identifying a particular pathway under certain conditions may be of practical importance in preventing the development of multidrug resistance.

## 4. The Effect of OS on Pgp and Role of the Transporter Protein during OS

### 4.1. The Effect of OS on Pgp Expression and Activity

A number of studies have evaluated the effect of OS on Pgp expression and activity. In a study by Ziemann et al. (1999) on a culture of rat hepatocytes, H_2_O_2_ at a concentration of 0.5–1 mM during incubation for 72 h was shown to increase the expression of the Pgp gene, its amount and its activity. The treatment of rat hepatocytes with catalase inhibitor 3-amino-1,2,4-triazole (2–4 mM for 72 h or 10 mM for 1 h) resulted in an elevated expression of *mdr1b* and Pgp mRNA. On the contrary, antioxidants (1 mM ascorbate; 10 mM mannitol) noticeably suppressed *mdr1b* mRNA expression and overexpression of Pgp. Furthermore, H_2_O_2_ activated poly(ADP-ribose) polymerase, a nuclear enzyme induced by DNA strand breaks, indicating that ROS reached the nuclear compartment [133].

The work of Felix and Barrand (2002) revealed that, in a primary culture of rat endothelium exposed to H_2_O_2_ at a concentration of up to 500 μM for 48 h, the expression of Pgp increased. Applying 100 μM H_2_O_2_ after 48 h caused a significant increase in Pgp functional activity, as assessed from [^3^H]vincristine accumulation experiments. Concentrations of more than 250 µM H_2_O_2_ decreased cell viability. At this concentration, H_2_O_2_, within 10 min, led to a transient increase in the level of intracellular reactive oxygen species (iROS), followed by its sustained decrease, detectable by flow cytometry [134]. 

At the same time, Hoshi et al. (2019) found that treatment of hCMEC/D3 cells (an in vitro blood–brain barrier model) with 0.5–5 mM H_2_O_2_ for a 20 min exposure reduced both Pgp efflux transport activity and protein expression on the plasma membrane, but not the protein expression in whole-cell lysate. The authors suggested that the rapid decrease in efflux function might be due to the internalization of Pgp. In the presence of dynasore (inhibitor of key molecule in endocytosis—dynamin), the decrease in Pgp protein in the plasma membrane fraction induced by 0.5 mM H_2_O_2_ was significantly suppressed [135]. 

Hydrogen peroxide increased the intracellular ROS level in a concentration-dependent manner (100–800 μM) after exposing retinal pigment epithelium D407 cells for 24 h. The Pgp expression in the mitochondria of D407 cells was increased, while antioxidants suppressed this process [136]. 

In a study on the culture of endothelial cells of cerebral vessels of rats, H_2_O_2_ at a concentration of 200 μM was shown to cause the development of OS (detected from the higher levels of dichlorofluorescein fluorescence) and to increase the expression of mRNA of the *mdr1a* and *mdr1b* genes, encoding Pgp, as well as the synthesis of the Pgp protein itself. The pretreatment of cells with polyethylene glycol catalase leveled out these changes [137]. 

Adding 1 μM of hydrogen peroxide for a period of 6 h to the culture medium of Caco-2 cells (a model of intestinal absorption of substances) increased the expression of Pgp in them, while the concentration of 10 mM led to a decrease in the expression of the transporter. The level of MDA, a reliable marker for lipid peroxidation, was increased in a concentration-dependent manner (1 and 10 mM) [138]. 

It was shown that the modeling of exogenous OS (adding H_2_O_2_ at concentrations of 0.1, 0.5 and 1 μM during incubation for 24 h and 10 μM for 72 h) on the cells of the Caco-2 line induced the expression and activity of Pgp [139]. The modeling of endogenous OS (inhibition of glutathione synthesis; DL-buthionine sulfoximine (BSO) at concentrations of 10, 50 and 100 μM; and an incubation period of 24 h) gave similar results [139]. The increase in ROS production in the used experimental models was detected by Mito-Tracker Red CM-H2XRos). 

At the same time, adding the antioxidant glutathione, along with the prooxidants, to the cells prevented the induction of Pgp under H_2_O_2_ action and reduced (100 μM) or suppressed (10 and 50 μM) it under BSO action. Partial suppression of Pgp induction under glutathione may take place since BSO, being a xenobiotic, can itself increase the amount of Pgp by stimulating the MDR1 gene expression [139].

Adding BSO to endothelial cells of rat brain microvessels at concentrations of up to 800 μM for 24 and 48 h caused an increase in protein Pgp and *mdr1a* and *mdr1b* mRNA expression. BSO, at a concentration of 200 μM, caused a significant increase in the functional activity of Pgp, which was evaluated in experiments on rhodamine 123 accumulation. In this model, a decrease in the concentration of intracellular glutathione and an increase in the ROS level were demonstrated; these effects were dependent on the time of exposure and BSO concentration. Concentrations of BSO equal to or greater than 400 μM reduced the cell viability. The ROS absorber N-acetylcysteine prevented ROS formation and attenuated changes in both the expression and Pgp activity induced by BSO [140]. 

Experiments in rats showed that 60 min of hepatic ischemia followed by 12 h of reperfusion increased plasma MDA levels. It also decreased the area under the pharmacokinetic concentration–time curve and the rate of enteral absorption of cyclosporine A (Pgp substrate), which may indicate the induction of functional transporter activity. Intravenous injection of an α-tocopherol analog, Trolox, 5 min before reperfusion reduced the degree of OS and inhibited Pgp expression [141]. In a study on Sprague-Dawley rats, feeding the rats with fructose with water at 10% wt./vol. for 8 weeks increased the level of substances, reacting with thiobarbituric acid in the left ventricle, and also reduced Pgp levels in cardiac tissue by ~ 30% compared to the control group [142].

The possible role of montelukast (ML), which is an antagonist of cysteinyl leukotriene receptors, in the fight against DOX-induced cardiotoxicity was studied [143]. Male Wistar rats were divided into five groups. The control group, the ML group, the DOX-treated group and the DOX/ML-treated groups received ML10 and 20 mg/kg/day for 14 days. In male Wistar rats, doxorubicin increased the level of cardiac enzymes in the blood serum, oxidative, inflammatory ((TNF-α)/survivin) and apoptotic ((IL)-1β/caspase-3 mRNA) markers, as well as the level of Pgp in cardiomyocytes. In the fight against DOX-induced cardiotoxicity, ML significantly improved the parameters of cardiac enzymes and weakened all parameters of OS; but at the same time, it increased the amount of Pgp both in comparison with the control and the isolated use of doxorubicin. Notably, ML, when used in isolation, did not affect the level of Pgp in cardiomyocytes [143].

Methotrexate (an effective chemotherapeutic drug against a wide range of tumors and autoimmune diseases) in rats caused the development of OS (there was an increase in the level of MDA, NO and SOD), an inflammatory reaction (iNOS and TNF-α) and damage to hepatocytes, and also reduced Pgp level. The preventive addition of a natural phenolic compound with antioxidant and anti-inflammatory characteristics, paeonol, reduced the severity of oxidative and inflammatory changes, which contributed to a decrease in Pgp levels [144]. In male rats, abamectin was shown to cause OS and to increase the expression of CYP-2E1, caspase-3 and p38 MAPK in the liver, as well as that of Pgp and GABA-A in the brain [145]. Thus, the same concentrations of the prooxidant H_2_O_2_ can have various effects on different cells, as they rely on various intracellular mechanisms.

Based on the analyzed works, the following conclusions could be drawn. 

It was shown during in vitro studies that very high prooxidant concentrations causing the development of OS contribute to a decrease in the expression and activity of Pgp, regardless of the duration of exposure. This fact may be due to the posttranslational modification of Pgp—the oxidation of amino acid residues in the transporter protein. For example, the most sensitive are SH groups, composed of cysteine and methionine, which account for 3.5% of the total number of amino acids in the Pgp structure. Moreover, in Pgp composition, amino acid residues of alanine, glycine, cysteine, tyrosine, etc., can be oxidized (Table 1). Likewise, a decrease in Pgp activity may be associated with cell damage (in most studies, when the Pgp activity decreased, cell viability also decreased). 

Moderate concentrations of prooxidants, not inflicting serious cell damage when exposed for 24 h or more, resulted in an increase in the expression of mRNA, protein, and Pgp activity. Sometimes, an increase in Pgp amount is not accompanied by that in the transporter activity, which is most likely implicated by the oxidative damage of Pgp molecule during the free radical production. 

In vivo studies yielded conflicting results. This may be due to the use of different OS models. For example, methotrexate can not only cause the development of OS but also reduce the expression of mRNA constitutive androstane receptor (CAR) and pregnane X receptor (PXR), which are Pgp activators [146]. At the same time, during ischemia, HIF1a is activated, which can increase the Pgp expression. 

### 4.2. Mechanisms of Changes in Pgp Expression and Activity during OS

The expression level of the *MDR1* gene plays a key role in the regulation of Pgp activity. It is initiated by a large number of stimuli that converge on a common region of the promoter called the “*MDR1* enhanceosome” [147]. The Pgp promoter contains binding motifs of stress-induced regulators of *MDR1* expression, including nuclear factor kappa-b (Nf-kB, nuclear-factor kappa light chain-enhancer of activated B cells) (from −167 to 158 bp) and p53 (from −72 to −40 bp).), HIF1 (−45 bp) [148,149,150]. In the Pgp promoter, there are also binding motifs of xenobiotic receptors: constitutive androstane receptor and pregnane X receptor (~8000 bp) [151,152]. 

The mechanisms of changes in the expression and activity of Pgp under OS were touched upon in several works. The incubation of a number of tumor cell cultures (KB31, KBV1, A549 and DMS-53) on hypo- and hyperglycemic media revealed the development of OS relying on NADPH-dependent oxidase 4 and the destabilization of mitochondrial membranes, activation of transcription factor HIF1 and an increase in its expression, nuclear translocation of the p65 subunit of transcription factor Nf-kB. There was also a significant activation of the functional activity of Pgp, leading to the development of cell resistance to doxorubicin (a transporter substrate) [153]. 

The treatment of HepG2 cell culture with a specific inhibitor of reduced glutathione synthesis—buthionine sulfoximine—for 12 h prior to exposure to hypoxic conditions led to a dose-dependent inhibition of HIF1a synthesis. Furthermore, an increase in the intracellular content of reduced glutathione by adding N-acetylcysteine to the culture medium partially restored the level of this transcription factor. In turn, the amount of HIF1a correlated with the level of Pgp [154].

HIF-1 is a polypeptide that is present in all cell type; it is responsible for compensatory and adaptive changes in the transcriptional activity of more than 100 human genes under conditions of oxygen deficiency. These changes include modulation of the synthesis of glucose transporters, erythropoietin, vascular endothelial growth factor, a number of glycolysis enzymes and the Krebs cycle; changes in angiogenesis and vascular tone; and regulation of hypoxia-induced apoptosis [155,156]. The transcription factor is a protein heterodimer that comprises two subunits: HIF-1α (120 kDa) and HIF-1β (or ARNT—aryl hydrocarbon nuclear translocator; 91–94 kDa). The HIF-1ß subunit is permanently located in the cell and is capable of forming heterodimers with various molecules, containing the bHLH-PAS domain. At the same time, the HIF-1α subunit is an obligatory oxygen-sensitive component of HIF-1: the expression of its mRNA, the half-life of the molecule and the activity of the transactivation domain are determined by the intracellular oxygen level [155,156,157]. Under hypoxic conditions, the HIF-1a subunit is stabilized and transported to the nucleus, where it forms a dimer with HIF-1β. It then binds to the DNA sites containing the so-called hypoxia–reactive elements, including hypoxia response element, HRE, as well as cis-active elements, namely transcription regulators (cis-acting transcriptional-regulatory element) of target genes [17]. In the MDR1 gene, this area is a complex of 5′-GCGTG-3′ nucleotides, located at a distance of −49 to −45 nucleotide pairs from the transcription start site [150,158].

We showed, for the first time, that the exposure of Caco-2 cells to hydrogen peroxide (5, 10 and 50 µM) for 72 h can increase the expression of the constitutive androstane receptor (CAR) [159]. In zebrafish embryos, mercaptopropionic acid salts MPACdTe and MPA-CdSCdTe were shown to cause OS, activate PXR and Nrf2 and slightly increase the expression of the *mdr1* gene mRNA [160]. We also showed that the product of OS— MDA—increases the amount and activity of Pgp (concentrations and timing). MDA at concentrations of 10 and 50 µM and an exposure duration of 24 h increased the relative amount and activity of Pgp, acting through CAR and pregnane X receptor (PXR). MDA can be transported by Pgp, which may have a protective and adaptive value [161].

CAR and PXR are protein transcription factors that recognize specific sequences in promoters or enhancers of target genes and modulate their expression. These transcription factors are considered to be xenobiotic sensors that have a regulatory effect on the transcription of drug transporter genes (including Pgp), as well as key metabolic enzymes [162,163]. CAR and PXR can interact with the responsive element (DNA sequences which interact with receptors) of the nuclear receptor in the area of 7.8 kilobase pairs of the *MDR1* gene enhancer and activate its expression through the DR4 motif, with which these receptors bind as a heterodimer to the retinoic acid receptor or as a monomer [164,165].

In an in vivo study of cerebral ischemia, induced by bilateral permanent occlusion of the common carotid arteries in Wistar rats, an inverse correlation between the Pgp expression in the blood–brain barrier and the level of free sulfhydryl groups in brain tissue was demonstrated [166]. With the help of specific inhibitors, the contribution of the main factors to the induction of Pgp under OS was evaluated. The important role of the Nrf2-Keap1 signaling pathway in increasing the Pgp amount under modeling OS caused by hydrogen peroxide was established. The transcription factor HIF1 participated in the regulation of the Pgp amount under 24 h exogenous OS, and the transcription factor CAR under 72 h OS [139]. Similarly, in adenocarcinoma human alveolar basal epithelial cells (A549) and colon carcinoma cells (HCT-116/R), NADPH oxidase isoform (NOX) 2 and Nrf2 were associated with the development of drug resistance due to the stimulation of *MDR1* expression, and they may be potential targets for overcoming drug resistance during cancer therapy [167,168].

Nrf2 is a redox-sensitive transcription factor reacting to changes in the ratio of reduced and oxidized SH groups in proteins. Its expression increases with the development of OS and aims at protecting the cell from the effects of free radicals [169,170]. Under normal conditions, this transcription factor forms a complex with the repressor protein Keap 1 (their binding is regulated by a number of protein kinases), which, on the one hand, promotes the ubiquitination and proteasomal degradation of Nrf2 (a prerequisite for this process is the presence of two cysteine residues in the Keap 1 molecule) and, on the other hand, prevents its penetration from the cytoplasm into the nucleus [171]. After activation, the Keap1-Nrf2 complex dissociates, and Nrf2 translocates into the nucleus, where it binds to antioxidant-response elements (AREs) and activates the transcription of protective enzymes and proteins.

Human retinal pigment epithelium cells (RPE D407) were exposed to an increasing concentration of H_2_O_2_ for 24 h, which increased the rate of cell death and induced expression of Pgp, as well as activation of Nf-kB and its translocation into the nucleus. Inhibition or suppression of Nf-kB led to a decrease in oxidation-induced Pgp expression [172]. On the contrary, when simulating sepsis-induced acute lung injury and acute kidney injury, treatment with tocilizumab, an interleukin 6 inhibitor, inhibited OS and apoptosis in lung and kidney tissues; and it significantly suppressed the activation of Nf–kB, weakening the JNK signaling pathway and significantly increasing the expression of Pgp both in pulmonary and renal tissues [173]. Rats with hyperammonemia were created by intraperitoneal administration of ammonium acetate (NH4Ac, 4.5 mmol/kg). A significant increase in Pgp function was observed in the hippocampus of rats. Meanwhile, these changes corresponded to an increase in the levels of mRNA and proteins Pgp. There was also a significant increase in the level of nuclear factor-kB (Nf-kB) p65. Primary cultured endothelial cells of rat brain microvessels (rBMEC) were used for in vitro study. The data showed that 24 h exposure to ammonia significantly increased the function and expression of Pgp in rBMEC, which was accompanied by activation of Nf-kB. Moreover, the changes caused by ammonia were reversed by an Nf-kB inhibitor [174].

Nf-kB belongs to the family of transcription factors regulating the immune response, inflammation, cell growth, differentiation and apoptosis. In the cytoplasm, Nf-kB is in an inactive state since it is associated with IkB inhibitor proteins. Upon phosphorylation of this complex, the proteasome proteolysis of IkB occurs, and free Nf-kB moves from the cytoplasm to the nucleus, where it binds to kB sites in the promoter area of genes and regulates their transcription [175]. Evidently, Nf-kB can bind the first intron of the *MDR1* gene promoter and increase its expression [176].

Sp1 (specificity protein 1) is a member of the transcription factor family that is involved in a variety of biological processes and plays an important role in the processes of cell growth, differentiation, apoptosis and carcinogenesis [177,178]. The promoter of the *MDR1* gene contains a binding area with factor sp1 [179], and the expression of Sp1 and Sp3 in cortical neurons in vitro is largely induced by OS, caused by glutathione depletion or H_2_O_2_ in these cells [180].

Diesel engine exhaust gas (DD) particles are the main component of urban air pollution. In isolated rat brain capillaries exposed to DD, increased expression and transport activity of the key drug outflow transporter, Pgp, was observed (the duration of exposure was 6 h, and the concentration was 5 μg/mL). The induction of Pgp was canceled out by blocking transcription, protein synthesis, inhibition of NADPH oxidase or pretreatment of capillaries with antioxidants (SOD and catalase), thus indicating the role of ROS in this process. The increased regulation of Pgp also stopped when the TNF-1 receptor (TNF-R1) was blocked, and this was not observed in experiments with capillaries of mice that were knocked out by TNF-R1. The inhibition of JNK, but not Nf-kB, blocked DEP-induced activation of Pgp, indicating the role of AP-1 in this signaling pathway [181]. It was shown that the *mdr1* promoter has an AP-1 binding site [182].

In a study on male Wistar rats, 15 mg/kg of doxorubicin administered intraperitoneally caused OS development in the liver, including an increase in the levels of MDA and total nitrite/nitrate, as well as a decrease in reduced glutathione and superoxide dismutase and an increase in liver tumor necrosis factor-alpha (TNF-α), interleukin-10 (IL-10), transforming growth factor-beta1 (TGF-β1) and mRNA of interleukin-1beta (IL-1beta) and interleukin-6 (IL-6). The preliminary administration of pregnenolone, a pregnane X receptor agonist, to rats counteracted these DOX-induced effects. Moreover, pregnenolone increased the expression of Nrf2, hemoxygenase-1 (HO-1) and Pgp in the liver and decreased Keap1, counteracting the effects of DOX. Furthermore, pregnenolone prevented the DOX-induced activation and nuclear translocation of Nf-kB and increased the cleavage of caspase-3 [183].

**Figure 4:**
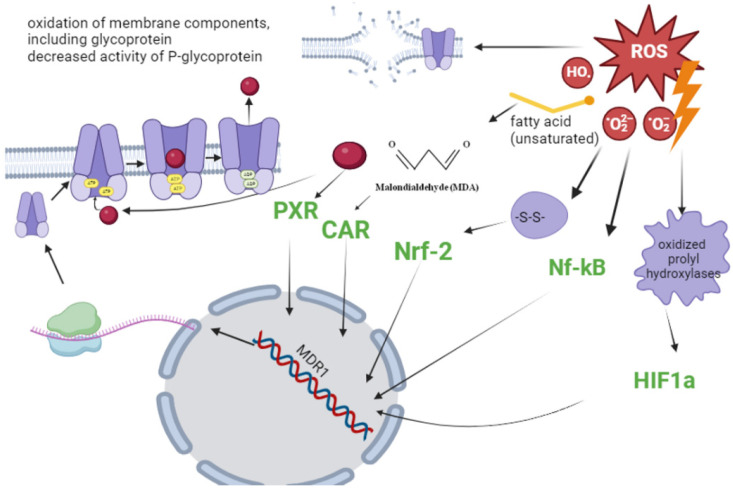
Mechanisms of Pgp regulation during OS (the figure was made using app.biorender.com, accessed on 2 October 2023).

A schematic representation of the main mechanisms of Pgp regulation during OS is presented in Figure 4.

### 4.3. The Role of Pgp during OS

Firstly, Pgp can protect the cell from prooxidants causing the development of OS, for example, the mitochondria of the oocyte exposed to nitrogen mustard gas [184].

In *MDR1*-deficient CD8+ T cells and intact CD8+ T cells, with the development of OS, the level of reduced glutathione decreased, and that of the oxidized glutathione dipeptide GSSG increased. On the contrary, only the wild-type cells were able to replenish reduced GSH levels over the next 12–36 h, and in *MDR1*-deficient CD8+ T cells, the level of reduced glutathione was not restored, and GSSG accumulated [185]. It was shown on retinal pigment epithelium cells (RPE D407) that a decrease in Pgp level by miRNA transfection further reduces the membrane potential of mitochondria and the rate of cell death under OS caused by H_2_O_2_ at 24 h of exposure [172].

Testing MDA for affiliation to Pgp substrates showed that, at a concentration of 10 μM, the transporter protein is involved in the transport of the peroxidation product. As the MDA concentration increased to 50 and 100 μM, Pgp’s contribution to MDA transport decreased, because its transport was apparently boosted by a passive diffusion [161]. The MDA molecule has a low molecular weight and can easily pass through a monolayer of cells [161,186].

Pgp induction was shown to increase Caco-2 cell survival under OS, whereas its inhibition reduced it [163]. Similar results were obtained with another peroxidation product—4-hydroxynonenal (4HNE)—and the MRP1 transporter protein (another member of ABC transporter superfamily) [187].

Thus, the currently available evidence suggests that Pgp plays a protective role during OS.

## 5. Conclusions

The currently available data strongly suggest that Pgp has a complex and diverse set of regulatory mechanisms working during OS. Moderate concentrations of prooxidants, not inflicting serious cell damage (eustress), increase Pgp expression and transporter activity. Of all the known mechanisms of Pgp regulation, the main role in OS is apparently played by the changes in the expression of mRNA *MDR1*. Some transcription factors may be involved in this process, the main ones being Nrf2 and Nf-kB. These factors often duplicate each other, and some can be activated under certain conditions, e.g., the deposition of oxidation products, depending on OS severity. Sometimes, an increase in Pgp expression is not accompanied by that of the transporter activity, which is most likely implicated by the oxidative damage of the Pgp molecule that happens during the free radical production. 

High prooxidant concentrations causing OS development (distress) contribute to a decrease in the expression and activity of Pgp, regardless of the duration of exposure. This fact may be due to the posttranslational modification of Pgp—the oxidation of amino acid residues in the transporter protein—or may be associated with cell damage. 

With in vivo studies, contradictory results have been obtained. This may be due to the use of different OS experimental models. 

Pgp plays a protective role during OS, as it eliminates the causative factors (prevents their penetration into cells), removes the products of OS from cells and participates in signaling pathways. 

At the moment, the role of ATP in the functioning of Pgp during the development of OS remains poorly understood, which is very important since mitochondria, the main producers of ATP, play a key role in this process. 

## Figures and Tables

**Table 1:** Oxidative modification of amino acid residues in the composition of Pgp.

Amino Acid in the Composition P-Glycoprotein	Quantity, pcs (Relative Content of the Total Amount, %)	Product of Oxidative Modification of the Amino Acid Residue
Leucine	120 (9.3)	Hydroperoxyleucine, hydroperoxyleucine
Alanine	118 (9.1)	Alanine Hydroxyperoxide
Isoleucine	105 (8.1)	Isoleucine hydroxyperoxide
Glycine	99 (7.7)	Glycine hydroxyperoxide
Serine	90 (6.9)	Glycolic aldehyde
Lysine	85 (6.5)	2-aminoadipine semialdehyde
Valine	83 (6.4)	β-hydroperoxyvalin, γ-hydroperoxyvalin
Glutamate	75 (5.8)	Pyruvic acid
Phenylalanine	70 (5.4)	2,3-dihydroxyphenylalanine, 4-hydroxyphenylalanine
Threonine	69 (5.3)	2-hydroxypropanal, acrolein
Arginine	64 (4.9)	Glutamic semialdehyde, 5-hydroxy-2-aminovaleric acid
Aspartate	57 (4.4)	Pyruvic acid
Asparagine	53 (4.1)	Carbonyl derivatives
Glutamine	53 (4.1)	Carbonyl derivatives
Methionine	38 (2.9)	Methionine sulfoxide, methionine sulfone
Tyrosine	35 (2.7)	3,4-dihydroxyphenylalaine, dityrosine, 3-nitrothyrosine, chlorthyrosine
Proline	31 (2.4)	Glutamic semi-aldehyde, 5-hydroxy-2-aminovaleric acid
Histidine	20 (1.6)	2-oxohistidine, 4-OH-glutamate, aspartate, asparagine
Tryptophan	18 (1.4)	Kynurenine, 3-hydroxynurenine, hydropyrolindole, oxindole, N- formylkynurenine
Cysteine	7 (0.5)	Thiol radicals, cystine, conjugates with glutathione sulfenic and sulfonic acids, nitrosothiols

## Data Availability

The data presented in this study are available upon reasonable request from the corresponding author.

## Supplementary Materials

The following supporting information can be downloaded at https://www.mdpi.com/article/10.3390/antiox13020215/s1, Figure S1: Primary amino acid sequence of human P-glycoprotein 1 (isoform encoded by the *ABCB 1* gene). This isoform is conventionally accepted as the canonical sequence of P-glycoprotein 1. NBDs sites binding ATP molecules are indicated in bold italics on a dark gray background. TM segments of the first TMD wagon train are marked with white letters on a light gray background. TM segments of the second TMD are indicated in ordinary bold on a dark gray background; nucleotide binding domains NBD1 and 2 are framed; glycosylation sites are indicated in bold italics on a dark gray background; and transmembrane domains (TMD1 and TMD2) are indicated by underscores https://www.uniprot.org/uniprotkb/P08183/entry, accessed on 6 September 2023.
