# Supplementary material for: Regulation of P-Glycoprotein during Oxidative Stress
Source: Antioxidants (Basel). 2024 Feb 8;13(2):215. doi: 10.3390/antiox13020215 (PMC10885963; doi:10.3390/antiox13020215)
Supplement: Supplementary file 1 [file antioxidants-13-00215-s001.zip › antioxidants-2813576-supplementary.pdf]

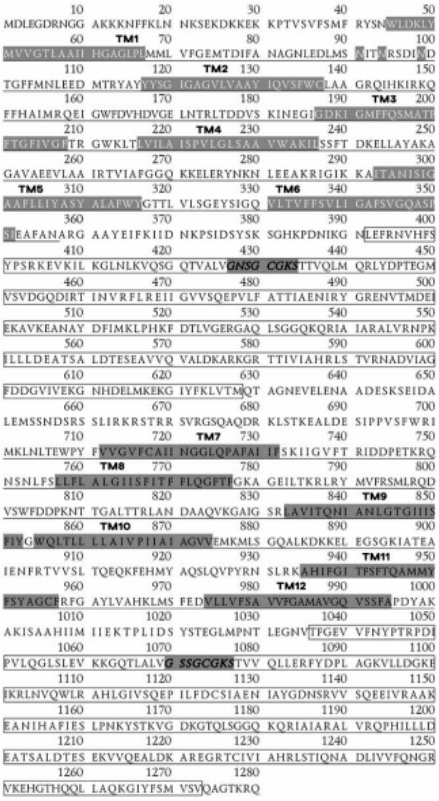

Figure S1. The following supporting information can be downloaded at www.mdpi.com/xxx/s1, Figure S1: Primary amino acid sequence of human P-glycoprotein 1 (isoform encoded by the *ABCB 1* gene). This isoform is conventionally accepted as the canonical sequence of P-glycoprotein 1. NBDs sites binding ATP molecules are indicated in bold italics on a dark gray background. TM segments of the first TMD wagon train are marked with white letters on a light gray background. TM segments of the second TMD are indicated in ordinary bold on a dark gray background; nucleotide binding domains NBD1 and 2 are framed; glycosylation sites are indicated in bold italics on a dark gray background; and transmembrane domains (TMD1 and TMD2) are indicated by underscores https://www.uniprot.org/uniprotkb/P08183/entry, accessed 6 September 2023.
